# Decreased CD57 expression of natural killer cells enhanced cytotoxicity in patients with primary sclerosing cholangitis

**DOI:** 10.3389/fimmu.2022.912961

**Published:** 2022-08-17

**Authors:** Bin Liu, Guo-Xiang Yang, Ying Sun, Takashi Tomiyama, Weici Zhang, Patrick S. C. Leung, Xiao-Song He, Sandeep Dhaliwal, Pietro Invernizzi, M. Eric Gershwin, Christopher L. Bowlus

**Affiliations:** ^1^ Division of Rheumatology, Allergy and Clinical Immunology, University of California at Davis, Davis, CA, United States; ^2^ Department of Rheumatology and Immunology, Affiliated Hospital of Qingdao University, Qingdao, Shandong, China; ^3^ Department of Liver Disease, Senior Department of Hepatology, The Fifth Medical Center of PLA General Hospital, Beijing, China; ^4^ Third Department of Internal Medicine, Division of Gastroenterology and Hepatology, Kansai Medical University, Osaka, Japan; ^5^ Division of Gastroenterology and Hepatology, University of California at Davis School of Medicine, Sacramento, CA, United States; ^6^ Division of Gastroenterology and Center for Autoimmune Liver Diseases, Department of Medicine and Surgery, University of Milan-Bicocca, Monza, Italy

**Keywords:** primary sclerosing cholangitis, cytotoxicity, CD57, nature killer cells, pathgenesis

## Abstract

**Background/aims:**

Primary sclerosing cholangitis (PSC) is a chronic inflammatory biliary disease for which the immunopathological basis remains an enigma. Natural killer (NK) cells are key components of innate immunity and seemingly play diversified roles in different autoimmune disorders (AIDs). The aim of this study was to determine the role of NK cells in the pathogenesis of PSC.

**Methods:**

The frequency and phenotype of circulating NK cells in a large cohort of patients with PSC and healthy controls (HCs) were systematically examined. In addition, the functional capacity of NK cells including cytotoxicity and cytokine production was studied.

**Results:**

The frequency of CD3^−^CD56^dim^CD16^+^ (defined as CD56^dim^) NK cells in PSC patients was significantly lower in comparison to HCs. CD56^dim^ NK cells from PSC displayed a more immature phenotype including high expression of the natural killing receptor NKp46 and downregulation of the highly differentiated NK cell marker CD57. Interestingly, the reduction of CD57 expression of NK cells was associated with the disease severity of PSC. In addition, PSC CD56^dim^ NK cells exhibited increased CD107a degranulation and cytolytic activity toward target cells compared with HCs. Further analysis demonstrated that CD57^−^CD56^dim^ NK cells from PSC had elevated expression of NKp46, NKp30, IL-2 receptor, and KLRG1 and higher cytotoxic capacity as compared to CD57^+^CD56^dim^ NK cells.

**Conclusions:**

Our data demonstrate that the differentiation of PSC NK cells is dysregulated with enhanced cytotoxic activity. This change is likely to be functionally involved in pathogenesis and disease progression, deducing the potential of NK-directed immunotherapy for PSC.

## Highlights

- The frequency of circulating CD56^dim^, but not CD56^bright^, NK cells in PSC is reduced; these cells show a greater ability of cytotoxicity, compared with HCs.
- The expression levels of CD57 on circulating CD56^dim^ NK cells positively correlate with the disease severity of PSC.
- CD57^−^CD56^dim^ NK cells, which express higher levels of natural cytotoxicity receptors NKp46 and NKp30, are potentially more cytotoxic than CD57^+^CD56^dim^ NK cells in PSC.
- The expression of CD57 on CD56^dim^ NK cells could be a new biomarker for monitoring disease progression and potentially for developing new NK cell-directed therapeutic strategies for PSC.

## Introduction

Primary sclerosing cholangitis (PSC) is a chronic autoimmune disorder of intra- and extra-hepatic bile ducts and is frequently associated with inflammatory bowel disease (IBD) (1, 2). Despite considerable data on the geoepidemiology, natural history, and genetic associations of PSC, the immunopathological mechanisms remain elusive. Natural killer (NK) cells are important effectors of the innate immune system and play a critical role in regulating adaptive immune responses through their ability to kill abnormal cells rapidly and produce a vast array of cytokines and chemokines. The cytotoxic function of NK cells is regulated through the opposite effects of inhibitory and stimulatory signals that result from interactions between NK receptors and target cell ligands (3, 4). Previous studies had suggested that NK cells were involved in the pathogenesis of PSC. Specific HLA class I variants, which were inhibitory ligands of killer-immunoglobulin receptors (KIRs) on NK cells, were reduced in frequency in patients with PSC (5). In contrast, activating ligands (including MHC class I chain-related gene A (MIC-A)), which could directly activate NK cell receptors, were significantly increased (6, 7). These data suggest that an imbalance of KIR and/or HLA class I ligands resulting in reduced inhibition or enhanced activation might be pivotal in PSC pathogenesis (5). Notably, the cytolytic activity of liver-derived NK cells from PSC was impaired due to the high local production of TNF-α (8). It has also been demonstrated that NK cells are increased in the colonic mucosa of patients with PSC (9). However, the exact mechanisms of how KIR/HLA class I ligand genotypes influence NK cell phenotype, susceptibility, and function and thus contribute to the development of PSC are unclear.

Human circulating NK cells could be categorized into different populations based on the expression of two surface markers, CD16 and CD56. The two major subsets are CD56^bright^ CD16^dim/−^ (defined as CD56^bright^) and CD56^dim^CD16^bright/+^ (defined as CD56^dim^) NK cells. Compared to CD56^bright^ NK cells, which show potential for cytokine secretion, CD56^dim^ NK cells have enhanced cytotoxicity associated with a mature differentiation state (10, 11). In this study, we compared the phenotypic and functional characteristics of NK cells in a cohort of patients with PSC and HCs. Our findings provide new information for understanding the role of NK cells in the pathogenesis of PSC, which might facilitate the rational development of immunotherapeutic strategies that target NK cells in PSC.

## Materials and methods

### Study subjects and peripheral blood mononuclear cell isolation

PSC (n = 45) was diagnosed according to internationally recognized diagnostic criteria (12, 13), and healthy controls (HCs) (n = 44) were enrolled for this study (**Table 1**). All subjects provided written informed consent, and the study was approved by the Institutional Review Board at the University of California, Davis, prior to initiation of the study. Peripheral blood mononuclear cells (PBMCs) were isolated from venous blood samples using Histopaque-1.077 (Sigma-Aldrich, St. Louis, MO, USA) density gradient centrifugation (14).

**Table 1:** General characteristics and clinical features of study subjects.

Clinical information*	PSC (n = 45)	HCs (n = 44)
Age	46.5 ± 16.3	55.2 ± 15.4
Gender (male/female)	26/19	23/21
Albumin, g/dl	3.5 ± 0.6	–
ALP (U/L)	423.6 ± 383.9	–
ALT (U/L)	71.2 ± 40.9	–
AST (U/L)	69.3 ± 43.7	–
Total bilirubin (mg/dl)	2.0 ± 2.7	–
INR	1.1 ± 0.2	–
WBC (K/mm^3^)	8.0 ± 3.3	–
Hematocrit (%)	39.8 ± 5.9	–
Platelet (K/mm^3^)	271.8 ± 166.8	–

PSC, primary sclerosing cholangitis; HCs, healthy controls; IBD, inflammatory bowel disease; ALP, alkaline phosphatase; ALT, alanine aminotransferase, AST, aspartate aminotransferase; INR, international normalized ratio; WBC, white blood cells.

*Data are expressed as mean ± SD.

### Cell staining and flow cytometry analysis

PBMCs were stained for cell surface and intracellular markers and were analyzed by flow cytometry (FCM) as we previously described (14). For cell surface staining, PBMCs were re-suspended in staining buffer (0.2% bovine serum albumin (BSA), 0.04% EDTA, and 0.05% sodium azide in phosphate-buffered saline (PBS)) and stained for 30 min at 4°C with cocktails containing combinations of fluorochrome-conjugated mAbs for the cell surface markers CD3, CD16, CD56, CD57, CD69, CD94, NKp30, NKp44, NKp46, NKp80, KLRG1 (BioLegend, San Diego, CA, USA), CD45RO, CD122, CCR7, NKG2A, NKG2C, and CD158a/h/g (eBioscience, San Diego, CA, USA). The cell markers for NK subsets are shown in **Supplementary Figure 1**. Isotype antibodies with matching conjugates (all from BioLegend) were used in parallel as negative controls. For intracellular cytokine staining, cells were re-suspended in culture medium RPMI-1640 with 10% fetal bovine serum (FBS) and stimulated at 37°C for 4 h with Leukocyte Activation Cocktail in the presence of BD GolgiPlug (BD Pharmingen, San Diego, CA, USA). The cells were stained for surface CD3, CD16, CD56, and CD57, fixed, permeabilized with BD Cytofix/Cytoperm Solution (BD Biosciences, San Diego, CA, USA), and then stained for intracellular IFN-γ (BioLegend). A fluorescence-activated cell sorting (FACS) with FCM (BD Immunocytometry Systems, San Jose, CA, USA) upgraded for the detection of five colors by Cytek Development (Fremont, CA, USA) was used to acquire data, which were analyzed by CellQuest PRO software (BD Immunocytometry Systems). Representative flow plots and histograms for analyzing surface marker expression on NK cells from a patient with PSC are shown in **Supplementary Figure 2**. Immunohistochemistry of CD56 and CD57 in PSC and HC liver tissues was performed to determine what kinds of NK cells are located in proximity to the bile ducts. The result shows that CD56^+^ NK cells are located in proximity to bile ducts rather than CD57 ones (**Supplementary Figure 3**).

### CD107a degranulation assay

The cytolytic activity of NK cells was evaluated by monitoring the surface expression of CD107a on NK cells upon contact with K562 target cells (15). Freshly isolated PBMCs were incubated with or without IL-2 (100 Unit/ml) at 37°C overnight. The cells (1 × 10^6^) were then mixed with K562 cells at a serial ratio (as indicated in the Results) in round-bottom 96-well plates and incubated with anti-CD107a mAb (BioLegend) and monensin (eBioscience) at 37°C with 5% CO_2_ for 4 h. For evaluation of NK cell degranulation by cross-linking of the receptor NKp46, these cells were incubated in an anti-NKp46 antibody (BioLegend) pre-coated 96-well plates for 4 h. After being washed with PBS–0.2% BSA, cells were stained with anti-CD3, anti-CD16, anti-CD56, and anti-CD57 mAbs. The frequency of CD107a-expressing CD3^−^/CD56^+^CD16^+^ NK cells was analyzed by FCM.

### Natural killer cell isolation and NKp46-mediated redirected killing assay

NK cells were purified from the PBMCs using a human CD56^+^CD16^+^ NK cell isolation kit, following the manufacturer’s recommendations (Miltenyi Biotec, Bergisch Gladbach, Germany). The purity of prepared NK cells was >95%. For NKp46-mediated redirected killing assay, purified NK cells were cultured with IL-2 (100 Unit/ml) overnight. The next day, NK cell target FcγR^+^P815 cells were labeled with lipophilic fluorochrome PKH-67 (Sigma-Aldrich, St. Louis, MO, USA) as previously described (16). The PKH-67^+^ P815 cells were loaded with anti-NKp46 antibody or IgG isotype (10 μg/ml; both from BioLegend) for 30 min at 4°C. After being washed, the antibody-loaded PKH-67^+^ P815 cells were co-incubated with purified NK cells at a 1:1 ratio for 4 h at 37°C. The cultured cells were stained with PE-Annexin V (BioLegend). The frequency of Annexin V positive PKH-67^+^ P815 cells was analyzed by FCM. Specific lysis was calculated as (% Annexin V^+^PKH67^+^ [dead targets] − % spontaneous Annexin V^+^PKH67^+^ [dead targets])/(100 − % spontaneous Annexin V^+^PKH67^+^ [dead targets]).

### Statistical analysis

The comparisons between the two groups were performed using the two-tailed unpaired Student’s *t*-test, non-parametric test, or two-tail paired t-test as indicated in the figure legends. Linear regression was used for correlation analysis. Data were expressed as mean ± standard error of the mean (SEM). p-Values smaller than 0.05 were considered statistically significant.

## Results

### CD56^dim^ natural killer cells are less frequent in peripheral blood mononuclear cells of primary sclerosing cholangitis

**Figure 1:**
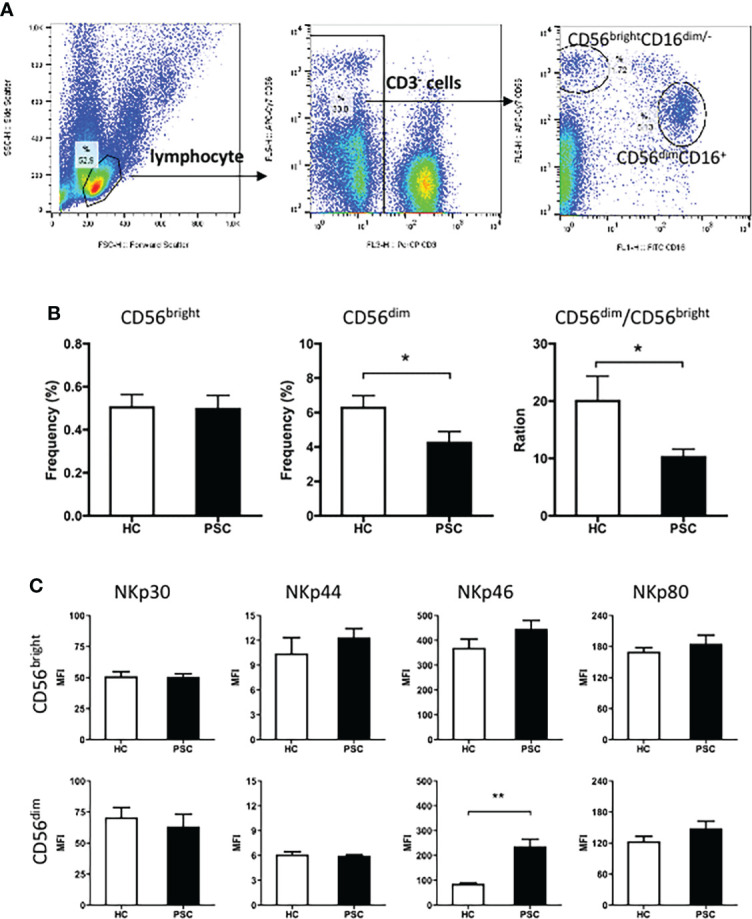
Characteristics of peripheral NK cells in patients with PSC. PBMCs from PSC and HCs were isolated and stained with fluorochrome-conjugated antibodies for cell subsets. **(A)** Strategy of FCM of NK cell subsets. CD3 and CD56 expression in PBMCs was analyzed after application of a lymphocyte gate. CD3-negative cells were further gated for analysis of CD3^−^CD56^bright^CD16^−^ (CD56^bright^) and CD3^−^CD56^dim^CD16^−^ (CD56^dim^) NK cell subsets. **(B)** The frequency (%) of CD56^bright^ and CD56^dim^ cells in total lymphocytes and **(C)** MFI of natural cytotoxicity receptors in each NK subset were compared between PSC (n = 34) and HCs (n = 29). *, p < 0.05; **, p < 0.01 (two-tailed Mann–Whitney test). NK, natural killer; PSC, primary sclerosing cholangitis; PBMCs, peripheral blood mononuclear cells; HCs, healthy controls; FCM, flow cytometry; MFI, mean fluorescence intensity.

Human NK cells can be categorized into different populations based on the relative expression of the surface markers CD16 and CD56. We first compared the frequencies of two major NK cell subsets CD56^bright^ and CD56^dim^ in the lymphocyte gate between PSC patients and HCs. The strategy of NK cell analysis by FCM is shown in **Figure 1A**. Compared to the HCs, CD56^dim^ NK cells were less frequent in lymphocytes from PSC patients, whereas the frequency of the CD56^bright^ NK cells was not significantly different (**Figure 1B**). In addition, a significantly lower ratio of CD56^dim^ to CD56^bright^ NK cells was observed in patients with PSC (**Figure 1B**), implying a significant reduction of CD56^dim^ NK cells in relation to the alteration of CD56^bright^ NK cells.

The natural cytotoxicity receptors were important activation receptors of NK cells. The expression of these receptors on each NK cell subset was therefore compared between PSC and HCs. The mean fluorescence intensity (MFI) of surface NKp30, NKp44, NKp46, and NKp80 was significantly higher on CD56^bright^ than CD56^dim^ NK cells in both PSC and HCs. Notably, compared to that from HCs, the expression of NKp46, but not other receptors that we measured, was significantly greater on CD56^dim^ NK cells from PSC (**Figure 1C**). In contrast, NKp46 expression on CD56^bright^ NK cells was not different between PSC and HCs.

**Figure 2:**
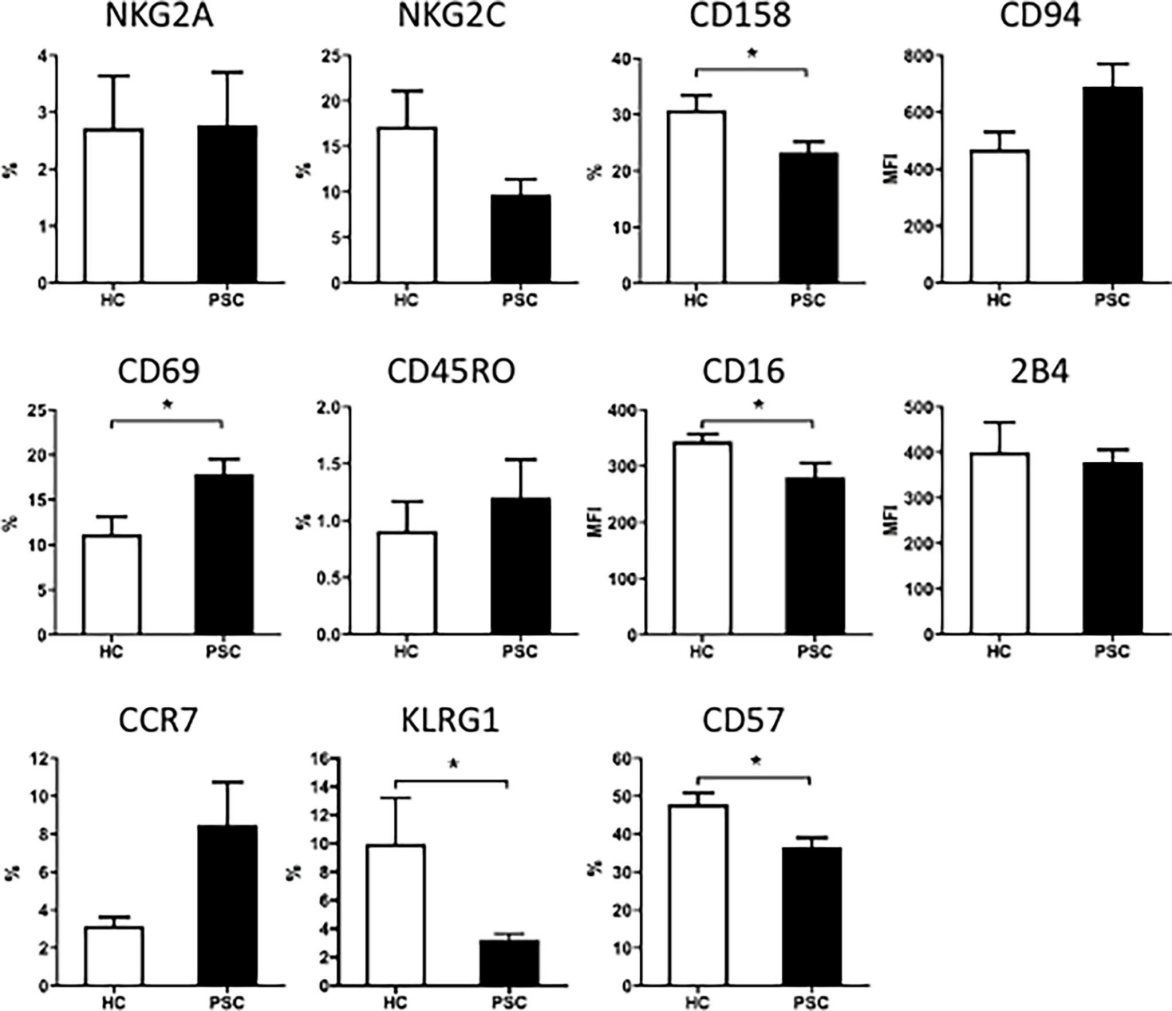
The expression of surface molecules on CD56^dim^ NK cells from patients with PSC. PBMCs were stained with fluorochrome-conjugated antibodies for different markers and analyzed by FCM. Percentage (%) of positive cells for each molecule marker in CD56^dim^ NK cells or MFI of each molecule were compared between PSC (n = 34) and HCs (n = 29). *, p < 0.05 (two-tailed unpaired t-test or non-parametric Mann–Whitney test). NK, natural killer; PSC, primary sclerosing cholangitis; PBMCs, peripheral blood mononuclear cells; FCM, flow cytometry; MFI, mean fluorescence intensity; HCs, healthy controls.

To further examine the phenotypic changes of CD56^dim^ NK cells in PSC, we analyzed the expression of inhibitory and activation receptors and other surface molecules that were involved in NK cell function (**Figure 2**). Significant differences were not detected between PSC and HCs CD56^dim^ NK cells in the expression of NKG2A, CD94, NKG2C, 2B4, CD45RO, and CCR7 (**Figure 2**). However, the expression of CD57, CD158a/h/g, KLRG1, and CD16 was significantly reduced in PSC compared with HCs. In addition, the expression of CD69 on CD56^dim^ NK cells was higher in PSC than in HCs (**Figure 2**). These data suggested that in addition to the decrease in frequency, the phenotypic pattern of circulating CD56^dim^ NK cells was also altered in PSC patients compared to HCs.

### Decrease in CD57 expression is associated with the low frequency of CD56^dim^ natural killer cells in primary sclerosing cholangitis

**Figure 3:**
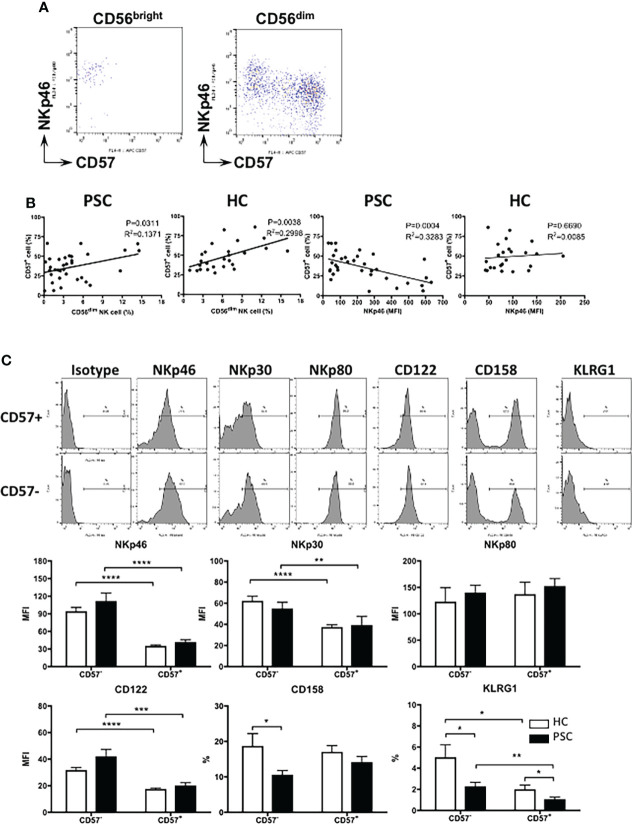
Phenotypic changes related to CD57 expression in PSC CD56^dim^ NK cells. **(A)** Pattern of NKp46 versus CD57 expression in CD56^bright^ and CD56^dim^ NK cell subpopulations. **(B)** Linear regression analysis for the correlation between the frequency of CD56^dim^ and CD57^+^ NK cells and between the MFI level of NKp46 and percentage of CD57^+^ cells in CD56^dim^ NK cells from PSC (n = 34) and HCs (n = 29). The p and r^2^ values are indicated in each graph. **(C)** Expression of different markers on CD57^−^ and CD57^+^ CD56^dim^ NK cells. NK cells from PSC (n = 17) were compared with HCs (n = 15). *, p < 0.05; **, p < 0.01; ***, p < 0.001; ****, p < 0.0001 (two-tailed unpaired t-test or non-parametric test for comparisons between PSC and HCs; two-tailed paired t-test or non-parametric test for CD57^−^ versus CD57^+^ NK cells in either patients or HCs). PSC, primary sclerosing cholangitis; NK, natural killer; MFI, mean fluorescence intensity; HCs, healthy controls.

CD57 has been suggested to be an intermediary marker in the differentiation of CD56^bright^ to CD56^dim^ NK cells (17). As shown in **Figure 3A**, CD57 is completely negative on CD56^bright^ NK cells from both PSC and HCs, whereas based on the expression of NKp46 and CD57, CD56^dim^ NK cells could be clearly divided into two differentiation subsets: NKp46^high^ CD57^−^ and NKp46^low/−^ CD57^+^ cells. Given that CD56^dim^ NK cells were less frequent in PSC than HCs (**Figure 1**), we analyzed whether this was associated with the lower CD57 expression on PSC NK cells. As shown in **Figure 3B**, the frequency of NKp46^low/−^ CD57^+^ NK cells positively correlated with the rate of CD56^dim^ NK cells in both PSC and HCs. Furthermore, the percentage of NKp46^low/−^ CD57^+^ cells was inversely correlated with NKp46 expression on CD56^dim^ NK cells from PSC, but not in HCs (**Figure 3B**). These data suggest that the extent of decreased CD57 expression is associated with the high level of NKp46 expression on circulating CD56^dim^ NK cells in PSC.

We further compared the expression of natural cytotoxicity receptors between NKp46^high^ CD57^−^ and NKp46^low/−^ CD57^+^ CD56^dim^ NK cells. NKp46 and NKp30, but not NKp80, were downregulated significantly from NKp46^high^ CD57^−^ to NKp46^low/−^ CD57^+^ CD56^dim^ NK cells in both PSC and HCs (**Figure 3C**). In addition, the expression of CD122 (IL-2 receptor β chain) and KLRG1 was also gradually decreased from CD57^−^ to CD57^+^ CD56^dim^ NK cells. We noted that the expressions of CD158a/h/g and KLRG1 were lower in PSC CD57^−^ CD56^dim^ NK cells than HCs, suggesting that reduced expression of CD57 is also associated with the alteration of CD56^dim^ NK cell phenotype in PSC.

### Reduced CD57 expression on CD56^dim^ natural killer cells correlates with disease severity of primary sclerosing cholangitis

**Figure 4:**
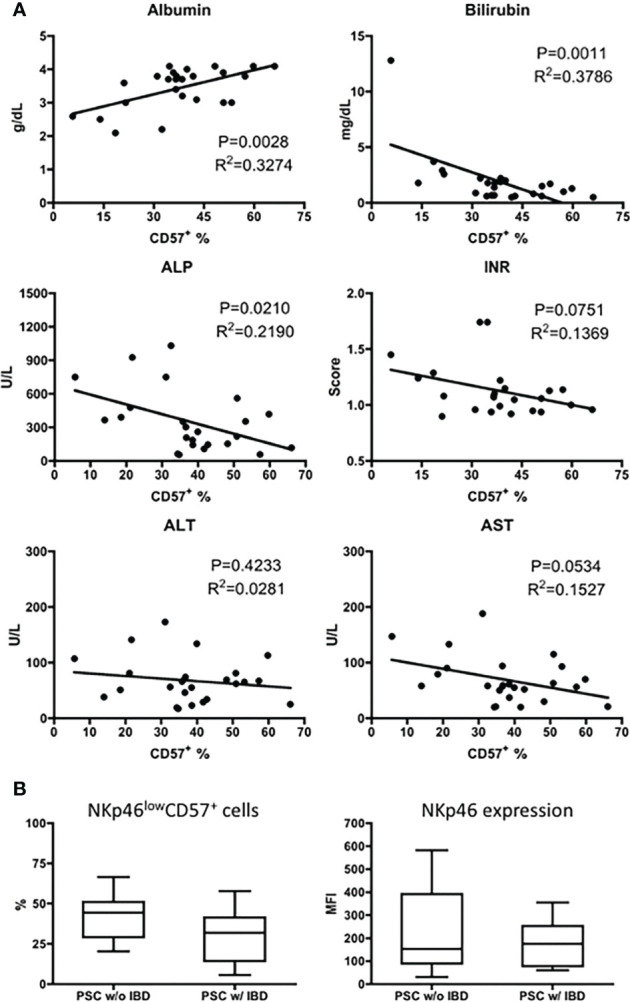
Correlation of CD57^+^CD56^dim^ NK cells with disease severity of PSC. **(A)** Scatter diagram showing the relationship between the frequency (%) of CD57^+^ cells in CD56^dim^ NK cells and the levels of biochemistry markers in patients with PSC (n = 25). The p and r^2^ values are indicated in each graph. **(B)** The expression of NKp46 (MFI) and CD57 (%) was analyzed in CD56^dim^ NK cells from PSC patients without IBD (PSC w/o IBD, n = 16) and those with IBD (PSC w/IBD, n = 17). NK, natural killer; PSC, primary sclerosing cholangitis; MFI, mean fluorescence intensity; IBD, inflammatory bowel disease.

To examine whether the downregulation of CD57 on CD56^dim^ NK cells was related to the disease severity of PSC, we compared the frequency of NKp46^low/−^ CD57^+^ NK cells with the patients’ biochemistry data. As shown in **Figure 4A**, the percentage of CD57^+^ CD56^dim^ NK cells positively correlated with the level of serum albumin and inversely correlated with the levels of bilirubin and alkaline phosphatase (ALP). A significant correlation was not detected between CD57^+^ CD56^dim^ NK cells and age (data not shown), alanine aminotransferase (ALT), aspartate aminotransferase (AST), or international normalized ratio (INR) (**Figure 4A**).

It has been demonstrated that increased expression of NKp46 on NK cells is involved in the pathogenesis of IBD (18). Since IBD is a common feature of PSC, we evaluated the expression of NKp46 on CD56^dim^ NK cells from PSC patients with or without IBD. As shown in **Figure 4B**, the MFI level of NKp46 and percentage of NKp46^low/−^ CD57^+^ NK cells are similar between PSC patients with and without IBD.

### Greater cytotoxicity of CD56^dim^ natural killer cells in primary sclerosing cholangitis compared with healthy controls in a redirected killing assay

**Figure 5:**
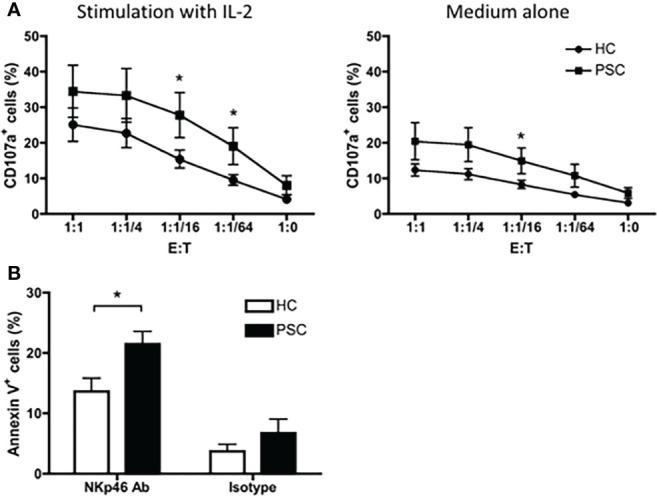
Cytotoxicity of CD56^dim^ NK cells. **(A)** CD107a degranulation in CD56^dim^ NK cells were compared between PSC (n = 12) and HCs (n = 13). PBMCs were cultured with or without IL-2 for 16 h. After being washed with culture medium, the cells were re-incubated with K562 cells at serial E:T (PBMC effector to K562 target) ratios in the presence of anti-CD107a antibody for 4 h. The percentages of CD107a^+^ cells in CD56^dim^ NK cells were analyzed by FCM. **(B)** NKp46-mediated NK cells redirected killing of P815 target cells by purified CD16^+^CD56^dim^ NK cells from PBMCs. Percentage of apoptotic Annexin V^+^ P815 cells was compared between PSC and HCs. *, p < 0.05 (two-tailed non-parametric Mann–Whitney test). NK, natural killer; PSC, primary sclerosing cholangitis; PBMCs, peripheral blood mononuclear cells; FCM, flow cytometry.

To address the functional characteristics of NK cells in PSC, we first performed a CD107a degranulation assay by *in vitro* incubation of PBMCs with the K562 target cell line. As shown in **Figure 5A**, a greater frequency of circulating CD56^dim^ NK cells from PSC expressed CD107a in comparison to NK cells from HCs with a wide range of effector—target ratios—suggesting that PSC CD56^dim^ NK cells were more cytotoxic. In addition, since PSC NK cells expressed higher levels of NKp46, we analyzed the NKp46-specific cytotoxic activity of purified CD56^dim^ NK cells in a redirected killing assay. As expected, PSC NK cells exhibited significantly greater NKp46-specific cytolytic activity against anti-NKp46 antibody-loaded p815 target cells than the NK cells isolated from HCs (**Figure 5B**). This difference was not observed when p815 target cells were loaded with an isotype control antibody, indicating the specific involvement of the NKp46 receptor in PSC NK cell cytotoxicity.

**Figure 6:**
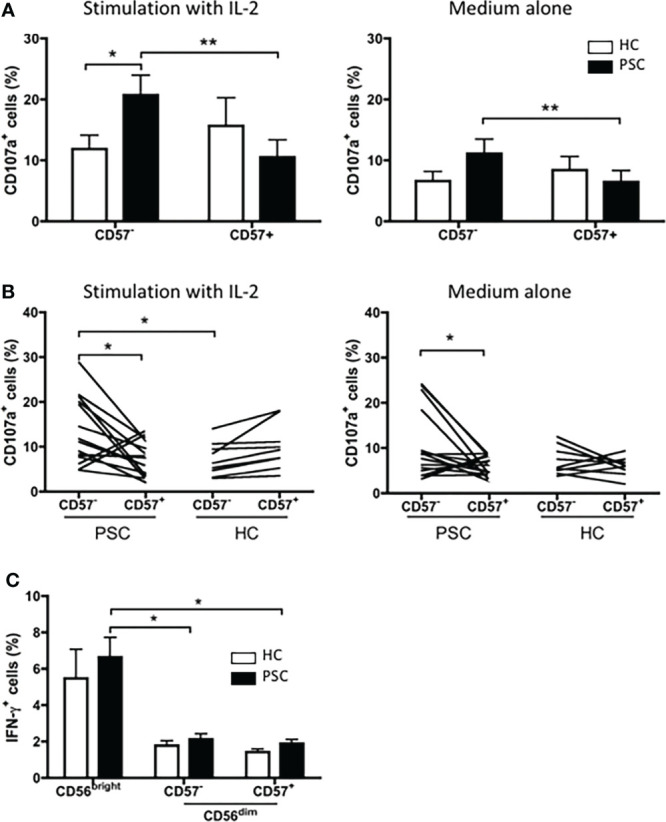
Cytotoxicity of CD57^+^ versus CD57^−^ CD56^dim^ NK cells in PSC. **(A)** PBMCs from PSC (n = 9) or HCs (n = 8) were co-incubated with K562 target cells at an E:T ratio of 1:1 in the presence of anti-CD107a antibody for 4 h. The percentages of CD107a^+^ CD57^+^ and CD107a^+^ CD57^−^ cells in the CD56^dim^ NK cell subset were compared. **(B, C)** PBMCs from PSC patients (n = 15) and HCs (n = 9) were cultured with the K562 target cells at an E:T ratio of 1:1 in an anti-NKp46 Ab-coated plate for 4 h. CD107a^+^ cells **(B)** and intracellular IFN-γ^+^ cells **(C)** in NK cells were analyzed by FCM. *p < 0.05; **p < 0.01 (two-tailed unpaired t-test or non-parametric test for comparisons between PSC and HCs; two-tailed paired t-test or non-parametric test for CD57^−^ versus CD57^+^ NK cells in either patients or HCs). NK, natural killer; PSC, primary sclerosing cholangitis; PBMCs, peripheral blood mononuclear cells; HCs, healthy controls.

We further compared the degranulation of CD56^dim^ NK cells between the NKp46^high^ CD57^−^ and NKp46^low/−^ CD57^+^ NK cell subpopulations. As shown in **Figure 6A**, the NKp46^high^ CD57^−^ cells had a significantly greater response to K562 target cells than NKp46^low/−^ CD57^+^ cells from patients with PSC. In contrast, there was no significant difference between NKp46^high^ CD57^−^ and NKp46^low/−^ CD57^+^ NK cells in the HCs. Furthermore, after activation by IL-2, NKp46^high^ CD57^−^ NK cells from PSC expressed higher levels of CD107a than those from HCs. We next examined whether activation of NK cells by cross-linking of NKp46 differentially affects the cytolytic function of the two CD56^dim^ NK cell subpopulations from PSC patients. Activation with antibody against NKp46 enhanced CD107a degranulation to a greater extent in NKp46^high^ CD57^−^ than in NKp46^low/−^ CD57^+^ CD56^dim^ NK cells from PSC, while there was no difference between the two NK cell subsets from HCs (**Figure 6B**). Particularly, NKp46^high^ CD57^−^ NK cells from PSC exhibited a higher level of CD107a expression than those from HCs after stimulation with IL-2 (**Figure 6B**). In contrast, the production of intracellular IFN-γ did not differ significantly between NKp46^low/−^ CD57^+^ and NKp46^high^ CD57^−^ NK cells in both PSC and HCs (**Figure 6C**), suggesting that activation of NK cells through the NKp46 signaling induced greater cytotoxicity, but not the production of IFN-γ in NKp46^high^ CD57^−^ NK cells from patients with PSC.

## Discussion

In this study, we examined the phenotypic characteristics and functional activities of NK cells in patients with PSC and demonstrated that the frequency of CD56^dim^, but not CD56^bright^ NK cells in lymphocytes, was significantly reduced in peripheral blood of patients with PSC compared to HCs. We further demonstrated that PSC CD56^dim^ NK cells appeared to be less differentiated, as these NK cells showed reduced expression of CD57 with highly expressing NKp46. CD57 is considered a marker for NK cells that are highly matured and terminally differentiated (19). The reduction of relatively differentiated CD57^+^CD56^dim^ NK cells in peripheral blood is significantly correlated with levels of several biochemistry markers linked to disease severity of PSC. Finally, we found that CD56^dim^ NK cells from PSC had greater cytotoxic activity than those from HCs, particularly in the redirected killing mediated through NKp46. Importantly, we noted that the CD57^−^ CD56^dim^ NK cells, which expressed high levels of NKp46, had more potential ability in cytotoxicity than CD57^+^ CD56^dim^ NK cells in PSC patients.

Analysis of NK cells in autoimmunity has shown that the frequencies or absolute numbers of circulating CD57^+^ NK cells are reduced in several autoimmune disorders (AIDs), suggesting that CD57^+^ NK cells play a regulatory role in the prevention and suppression of AID (20). In this study, we demonstrated that CD57^+^ CD56^dim^ NK cells were less frequent in PSC. Surprisingly, we found that in PSC patients, the NKp46^high^ CD57^−^ CD56^dim^ NK cells were more cytotoxic than NKp46^low/−^ CD57^+^ CD56^dim^ NK cells, whereas in HCs, such difference was not detected, even when the NK cells were stimulated with IL-2 (**Figure 6**). We propose that the imbalance of CD57^+^ to CD57^−^ in CD56^dim^ NK cells leads to stronger cytotoxicity of the NK cell population from PSC patients than those from HCs (**Figure 5**). We note that in target tissues, NK cells tend to be CD57^−^ with a higher turnover and degranulation ability, thus contributing to tissue damage (**Supplementary Figure 3C**) (4, 21). The levels of CD57 expression are lower in intrahepatic NK cells than those in the periphery (22, 23). It has been demonstrated that CD56^dim^ hepatic NK cells are not original tissue-resident but retain the homing feature and phenotype of peripheral CD56^dim^ NK cells, indicating that these cells can migrate from the periphery into the liver (24). Thus, dysregulation of CD57 expression in NK cells and accumulation of cytotoxic CD57^−^ CD56^dim^ NK cells in the liver-injury sites may lead to biliary cell damage in PSC (**Supplementary Figure 3D**).

We also demonstrated herein that the expression of NKp46, but not other NK receptors, was significantly increased in CD56^dim^ NK cells of PSC patients compared with HCs. Further analysis indicated that the NKp46 expression gradually decreased from CD56^bright^ to CD57^−^CD56^dim^ to CD57^+^CD56^dim^ NK cells (**Figure 3A**). Since there is no significant difference in NKp46 expression on either CD57^+^ or CD57^−^ NK cell subsets between PSC and HCs (**Figure 3C**), the higher expression of NKp46 in PSC CD56^dim^ NK cells than in HCs is likely due to the reduced frequency of NKp46^low/−^ CD57^+^ NK cells. Indeed, the expression of NKp46 is inversely correlated to the CD57 expression in PSC CD56^dim^ NK cells (**Figure 2**). It should be noted that the activation receptor NKp46 is essential for the *in vitro* functionality of NK cells (25) and the density of NKp46 expression correlates with NK cell cytolytic activity (26, 27). Herein, we demonstrated that NKp46 engagement by P815 target cells led to a higher level of degranulation of PSC NK cells compared with HCs (**Figure 5**), suggesting that NKp46 was crucial to the *in vivo* activity of NK cells against target cells. Recent studies also indicated that NKp46^high^ NK cells, which included both CD57^−^ CD56^bright^ and CD57^−^ CD56^dim^ cells, had higher cytolytic activity and IFN-γ secretion than NKp46^dim^ NK cells, which were mostly CD57^+^ cells. Importantly, the numbers of this NKp46^high^ NK subpopulation have been closely correlated with the severity of liver inflammation and damage in hepatitis C virus (HCV) patients (28, 29). In addition, NKp46-expressing NK cells had also been implicated in IBD (30, 31). IFN-γ-producing NKp46^+^CD56^+^ NK cells were shown to be significantly increased in lamina propria lymphocytes in patients with IBD (18). In a mouse model of colitis, IL-15-dependent NKp46^+^ cells amplified intestinal inflammation *via* the recruitment of inflammatory monocytes (30). In the current study, we demonstrated that the NKp46 expression was increased significantly in circulating CD56^dim^ but not CD56^bright^ NK cells from PSC patients compared with HCs. We further dissected the CD56^dim^ NK cells based on NKp46 and CD57 expression and found that NKp46^high^CD57^−^ NK cells had more potent lytic activity than NKp46^low^CD57^+^ CD56^dim^ NK cells. NKp46 engagement enhanced the degranulation of NKp46-highly expressing CD57^−^ NK cells from PSC (**Figure 6B**). Interestingly, a recent study of intrahepatic non-parenchymal cells from PSC livers demonstrated that a higher frequency of NKp46^+^ cells was associated with advanced fibrosis (32). Thus, despite the reduced circulating CD56^dim^ NK cells, a relatively high frequency of NKp46^high^CD57^−^ cells might still contribute to the pathogenesis of PSC. Accumulation of NKp46^high^CD57^−^CD56^dim^ NK cells in the livers of PSC patients and their engagement with activating ligands might render them more cytotoxic toward the biliary epithelial cells (28).

The expression of self-MHC-specific inhibitory receptors (e.g., KIR, LIR-1, or NKG2A) by NK cells correlated with a higher ability to kill target cells and produced IFN-γ in response to stimulation *via* activating receptors (33–35). Herein, we demonstrated that CD158 molecules (including KIR2DL1/S1/S3/S5) reduced expression in PSC NK cells compared to HCs, while there was no significant difference in the expression of inhibitory receptors NKG2A and CD94. In addition, the expression of CD158 was similar between CD57^+^ and CD57^−^CD56^dim^ NK cells in both PSC and HCs (**Figure 3**). We noted that the frequencies of HLA-Bw4 and HLA-C2, which were ligands for the inhibitory KIRs 3DL1 and 2DL1, respectively, were significantly reduced in PSC patients as compared with HCs (5).

As mentioned above, CD56^dim^ NK cells migrate from the periphery into the liver rather than the original liver-resident one. Meanwhile, it is so hard to obtain tissues sample from PSC patients. From this point of view, we think that the number of CD56dim NK cells in peripheral blood could reflect the intrahepatic situation of such cells in the liver of PSC patients to some extent. Hence, circulating NK cells of PSC patients are applied in the search. As a limitation of our research and technical challenges, further studies of the expression of inhibitory and activation KIRs in CD57^−^ *vs* CD57^+^CD56^dim^ NK cells are likely to reveal how these signals regulate NK cell differentiation. We also admit that the single-cell sequencing studies of intrahepatic and peripheral NK cells from patients with PSC will provide direct evidence support for elucidating the role of NK cells in the development of PSC.

In summary, our results suggest that in patients with PSC, deficiency in maturation and differentiation with aberrant expression of NKp46 and loss of CD57 is associated with enhanced cytotoxicity in the CD56^dim^ NK subset. This distinct population of NK cells could be functionally involved in the pathogenesis of PSC. Our findings deduce the potential for NK cell-specific immunotherapy for PSC.

## Data availability statement

The original contributions presented in the study are included in the article/**Supplementary Material**. Further inquiries can be directed to the corresponding authors.

## Ethics statement

This study was reviewed and approved by Institutional Review Board at the University of California, Davis. The patients/participants provided their written informed consent to participate in this study.

## Author contributions

BL and YS for Writing. G-XY and CB for Data curation, Funding acquisition and Methodology. TT, WZ, PL, X-SH for Formal analysis and Methodology. SD, PI and MG for Investigation. All authors contributed to the article and approved the submitted version.

## Funding

This study was funded by PSC Partners Seeking a Cure (2014).

## Acknowledgments

We thank Chung Heng Liu for preparing the blood samples and collecting the clinical information of patients.

## Conflict of interest

The authors declare that the research was conducted in the absence of any commercial or financial relationships that could be construed as a potential conflict of interest.

## Publisher’s note

All claims expressed in this article are solely those of the authors and do not necessarily represent those of their affiliated organizations, or those of the publisher, the editors and the reviewers. Any product that may be evaluated in this article, or claim that may be made by its manufacturer, is not guaranteed or endorsed by the publisher.

## Supplementary material

Supplementary Figure 1 Schematic of cell marks for NK subsets: Immature NK cells express CD56^bright^, absent, or CD16^dim^, low KIR, and CD27 and exhibit low cytotoxicity. Mature NK cells, in contrast, express CD56^dim^, high CD16, high KIRs, and CD57 and exhibit high cytotoxicity. CD57-CD56^dim^ NK cells, which express higher levels of natural cytotoxicity receptors NKp46 and NKp30, are potentially more cytotoxic than CD57+CD56^dim^ NK cells in PSC.

Supplementary Figure 3 H&E staining and immunohistochemistry of PSC patient liver tissue sections. A. Fibrosis and inflammation are observed in the bile ducts, along with hyperplasia and the typical features of “onion skin” fibrosis and fibrous obliterative cholangitis. B.CD3^+^ T cells in PSC patient liver tissue. C. CD57 is absent. D. CD56^+^ NK cells are located in biliary cell, and biliary epithelium is positive for CD56 in PSC patient. E-H. H&E staining and immunohistochemistry of HC.

## Abbreviations

PSC, primary sclerosing cholangitis; HCs, healthy controls; AID, autoimmune disorder; IBD, inflammatory bowel disease; PBMC, peripheral blood mononuclear cell; FCM, flow cytometry; MFI, mean fluorescence intensity; ALP, alkaline phosphatase; ALT, alanine aminotransferase; AST, aspartate aminotransferase; INR, international normalized ratio; WBC, white blood cells; NK, natural killer; KIRs, killer-immunoglobulin receptors; MIC-A, MHC class I chain-related gene A.
