# Supplementary figures and images for: Decreased CD57 expression of natural killer cells enhanced cytotoxicity in patients with primary sclerosing cholangitis
Source: Front Immunol. 2022 Aug 17;13:912961. doi: 10.3389/fimmu.2022.912961 (PMC9434697; doi:10.3389/fimmu.2022.912961)

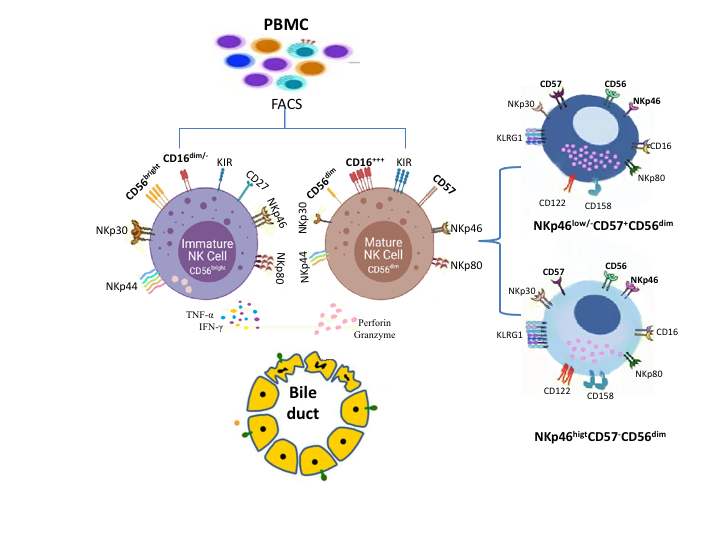

Supplement: Supplementary Figure 1 — Schematic of cell marks for NK subsets: Immature NK cells express CD56bright, absent, or CD16dim, low KIR, and CD27 and exhibit low cytotoxicity. Mature NK cells, in contrast, express CD56dim, high CD16, high KIRs, and CD57 and exhibit high cytotoxicity. CD57-CD56dim NK cells, which express higher levels of natural cytotoxicity receptors NKp46 and NKp30, are potentially more cytotoxic than CD57+CD56dim NK cells in PSC. [file Image_1.tiff]

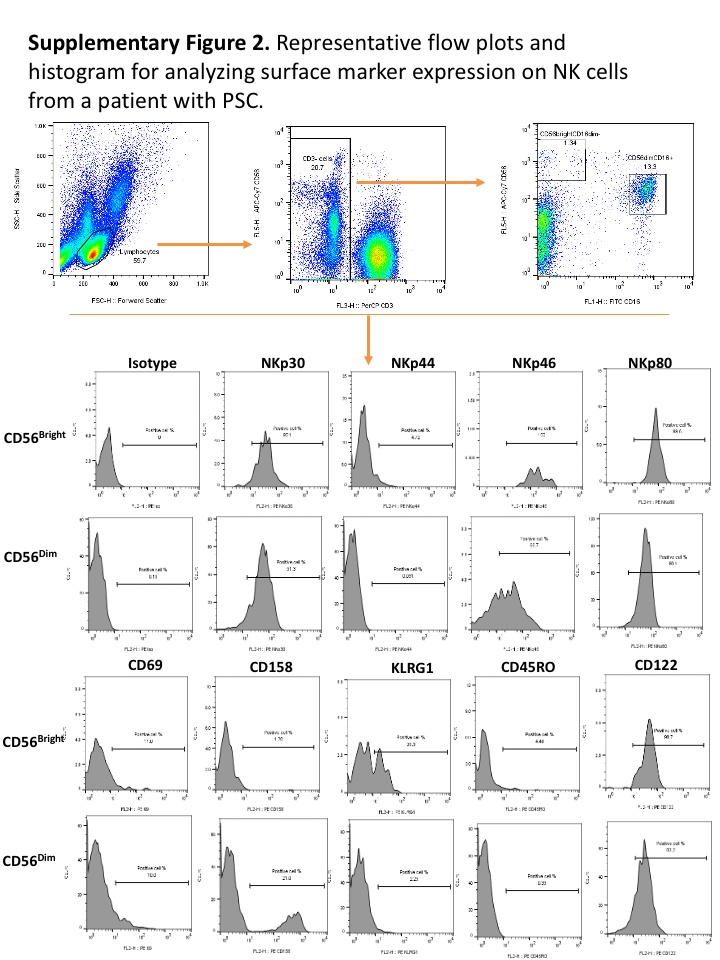

Supplement: Supplementary file 2 [file Image_2.tiff]

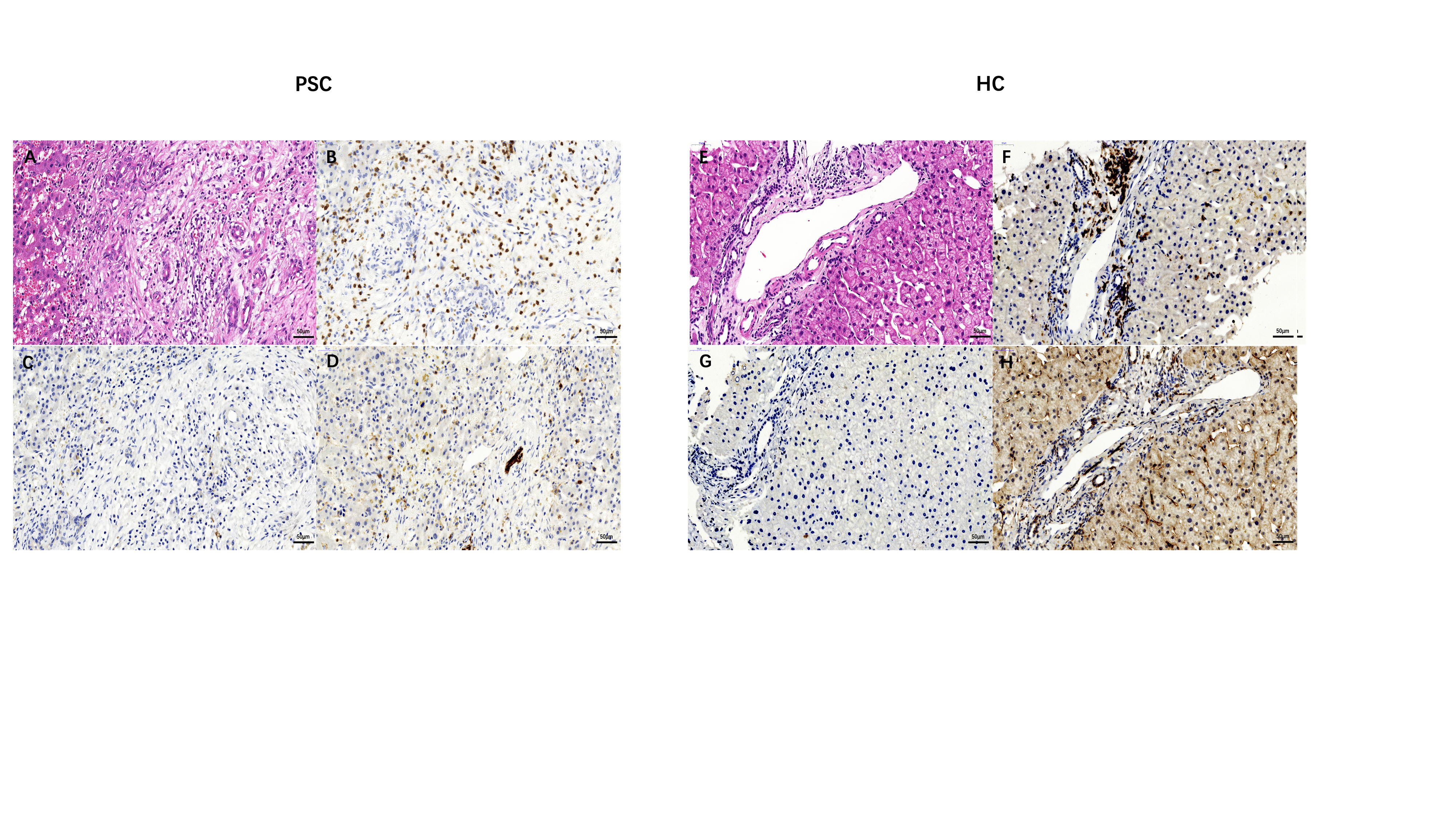

Supplement: Supplementary Figure 3 — H&E staining and immunohistochemistry of PSC patient liver tissue sections. A. Fibrosis and inflammation are observed in the bile ducts, along with hyperplasia and the typical features of “onion skin” fibrosis and fibrous obliterative cholangitis. B.CD3+ T cells in PSC patient liver tissue. C. CD57 is absent. D. CD56+ NK cells are located in biliary cell, and biliary epithelium is positive for CD56 in PSC patient. E-H. H&E staining and immunohistochemistry of HC. [file Image_3.tiff]
